# Examining attributes of retailers that influence where cannabis is purchased: a discrete choice experiment

**DOI:** 10.1186/s42238-023-00204-w

**Published:** 2024-02-08

**Authors:** Jennifer R. Donnan, Molly Downey, Karissa Johnston, Maisam Najafizada, Lisa D. Bishop

**Affiliations:** 1 School of Pharmacy, Memorial University of Newfoundland School of Pharmacy, 300 Prince Phillip Drive St. John’s, NL A1B 3V6 St. John’s, Canada; 2 Faculty of Science, Department of Psychology, Memorial University, St. John’s, NL Canada; 3 Faculty of Medicine, Memorial University of Newfoundland, St. John’s, Canada

**Keywords:** Cannabis, Consumer preferences, Discrete choice experiment, Cannabis retailer

## Abstract

**Background:**

With the legalization of cannabis in Canada, consumers are presented with numerous purchase options. Licensed retailers are limited by the Cannabis Act and provincial regulations with respect to offering sales, advertising, location, maximum quantities, and information sharing in an effort to protect public health and safety. The degree these policies influence consumer purchase behavior will help inform regulatory refinement.

**Methods:**

A discrete choice experiment within a cross-sectional online survey was used to explore trade-offs consumers make when deciding where to purchase cannabis. Attributes included availability of sales/discounts, proximity, product information, customer service, product variety, and provincial regulation. Participants ≥ 19 years old who lived in Canada and purchased cannabis in the previous 12 months were recruited through an online market research survey panel. A multinomial logit (MNL) model was used for the base model, and latent class analysis was used to assess preference sub-groups. Key limitations included ordering effect, hypothetical bias, and framing effect.

**Results:**

The survey was completed by 1626 people, and the base model showed that customer service carried the most weight in purchase decisions, followed by proximity and availability of sales and discounts. There was considerable heterogeneity in preference patterns, with a five-group latent class model demonstrating best fit. Only one group (15% of sample) placed a high value on the store being provincially regulated, while three groups were willing to make a trade-off with regulation to access better customer service, product information, or closer proximity. One group preferred non-regulated sources (24% of sample); this group was also primarily driven by the availability of sales and discounts. Three groups (60.5% of sample) preferred online stores.

**Conclusion:**

This study highlighted that there exists significant diversity with respect to the influence of consumer experiences on cannabis purchase behaviors. Modifications to cannabis retail regulations that focus on improving access to product information as well as reviewing limitations on sales and discounts could have the most impact for shifting customers to licensed retailers.

**Supplementary information:**

The online version contains supplementary material available at 10.1186/s42238-023-00204-w.

## Introduction

Cannabis was legalized for non-medical use in Canada in 2018 to protect public health and safety by providing regulated access to a safe cannabis supply [1]. A particular goal of this legislation was to reduce the unregulated cannabis market and associated illegal activities as well as to protect the public from potentially unsafe products. Despite the efforts put forth by the Canadian government, the results of the 2021 Canadian Cannabis Survey indicated that 37% of respondents purchased cannabis from non-legal sources at least some of the time [2]. Statistics Canada reported that as of 2019, cannabis purchases from unregulated sources accounted for 41.1% of total sales [3]; however, the true number is hard to gauge. Further shifting cannabis sales to the legal market will require a thorough understanding of the factors influencing consumer purchasing behaviors.

Cannabis products purchased through unlicensed sources are not regulated and are therefore not tested for accuracy of contents or presence of contaminants such as bacteria, pesticides, lead, or arsenic [4]. It has also been determined through laboratory testing that package labels may be inaccurate [5, 6], leading cannabis users to consume more or less than their desired potency or potentially experience adverse health effects. Additionally, the emergence of E-cigarette- or *vaping- use-associated lung injury* (EVALI) in 2019 was found to be associated with vitamin E acetate, an additive that has been detected in vape pens and cartridges, mostly from the unlicensed market [7, 8]. Given the potential health risks of unregulated cannabis products, additional efforts to encourage purchases through regulated sources can improve cannabis-related public health and safety in Canada’s legalized environment.

The 2021 Canadian Cannabis Survey [2] asked respondents to rank the relative importance of attributes that influenced their cannabis purchasing decisions. While the price was most commonly ranked as the primary factor, it was only identified as the most important factor 28% of the time. A close 26% of respondents named safe supply as the most important factor influencing purchasing, while others prioritized attributes such as quality, convenience, and regulation of cannabis source. Qualitative work conducted by our team to inform this study revealed that it was not only attributes of the product but also attributes of the retailers that influence consumer choices [9]. Even if product price, quality, and selection were mirrored between the licensed and unlicensed market, the customer experience can also play a role in where people make their purchases.

Cannabis consumers value the ability to gather information from knowledgeable staff to inform their purchasing decisions [9]. This includes information on the product itself such as its contents and growth and cultivation history as well as the experiences they might have while consuming the cannabis for both medical and non-medical purposes. Other retailer characteristics that consumers consider include those related to accessibility such as store proximity, hours of operation, and product variety, as well as store look or atmosphere and the availability of product sales and discounts [9]. Little research has been conducted regarding consumer preferences for retailer characteristics, especially in a context that can be generalized to the Canadian cannabis market. One discrete choice experiment (DCE) comparing big box supermarkets to family-owned grocery stores found that customer service and ambience played a role in decisions [10]. Another study found that proximity was important but that many factors influenced its relative influence, including possession of a driver’s license, number of accompanying persons, and sociodemographic characteristics [11]. There have been no studies to date that explore the relevant retailer attributes specific to cannabis purchasing decisions.

Consumers often make trade-offs between cannabis price and other important attributes, a behavior that can be explained by multi-attribute utility theory [12]. Multi-attribute utility theory posits that individuals make choices by considering a variety of aspects that will influence their satisfaction with a decision. People then make trade-offs between factors in order to produce the outcome that will give them maximum reward. The attributes that contribute to a decision and their relative importance vary between individuals and circumstances. One valuable tool for determining the attributes that contribute to decision-making behavior is the DCE. In a DCE, a respondent is presented with a series of hypothetical choices and instructed to select the option that they prefer [13]. Each option in a choice task involves different combinations of attributes which contribute to the respondent’s perceived utility of that choice. Through repeated trials, the individual’s responses reveal the trade-offs that they will make for particular attributes, indicating in turn the attributes most essential to their satisfaction.

From the perspective of cannabis purchasing, a consumer may trade-off the price in favor of superior customer service or greater product selection. As suggested by multi-attribute theory, the factors influencing cannabis purchase decisions vary from person to person [2]. The purpose of this study is to estimate the relative importance of attributes of cannabis retailers and to measure the trade-offs consumers make between those attributes.

## Methods

This study examined the preferences of cannabis consumers across Canada using a DCE within a cross-sectional online survey. The study was conducted in compliance with the general framework for good research practices regarding the use of DCEs by the International Society for Pharmacoeconomics and Outcomes Research [14].

### Participants

Individuals who lived in Canada, were aged 19 or older, and reported having purchased cannabis during the last 12 months were eligible to complete the survey. The age of 19 was chosen as this is the legal age to purchase cannabis in 11 of the 13 provinces and territories in Canada. The exception being 18 years in Alberta and 21 years in Quebec. A market research company (Angus Reid) was contracted to recruit a representative sample using their proprietary panel. The survey instrument, created and administered using Qualtrics, and consent was confirmed before proceeding to the survey questions. Respondents were offered points for survey completion which can be collected and exchanged for merchandise or gift cards.

### Study design

This study is part of a series of studies examining consumer choice for various types of cannabis products. This included a systematic review of the literature and qualitative interviews and focus groups [9, 15]. These were completed prior to the current study to establish attributes that influence cannabis consumer preferences. The DCE was part of a larger survey that included four unique DCE questions; the others included cannabis purchase preferences for dried flower [16], vapes [17], and edibles. While only respondents who indicated experience with purchasing dried flower, vapes, or edibles were presented with those DCE questions, all respondents were directed to respond to the question pertaining to retailers. A detailed protocol for this survey is available as a supplementary appendix in a previously published paper [17].

Attributes and levels that are relevant to both consumers and policy were determined based on data collected from the literature review an qualitative data collection [9] (Table 1). The attributes examined were as follows: availability of sales and discounts, store proximity, customer service, product information, product variety, and provincial regulation. These were prioritized based on importance portrayed by consumers in the qualitative interviews, as well as consideration for attributes that have policy implications. Initial versions of the attributes and levels were also presented to a group of stakeholders including a cannabis regulator, retailer, and consumer for input prior to finalization. The availability of sales and discounts included things like weekly sales, bulk pricing, free product with purchase, and loyalty programs. These enticing offers were mentioned frequently in qualitative interviews, specifically in relation to unlicensed sources as there are strict regulations on advertising and promotion within the Cannabis Act [18]. Store proximity captured both physical distance to a brick-and-mortar store as well as online sales with home delivery. Customers we spoke with had different behaviors and preferences with online vs. in person stores. And while physical distance came up less frequently, there are jurisdictional regulations that control store density, and understanding the role proximity places in purchase decisions could inform such regulations. Our focus groups and interviews highlighted how much consumers value access to information and good customer service. There are issues around customer service related to the ability of a store employee to be able to answer questions and support product selection. Product information was focused on access to information on packages or other company pamphlets or materials available in the store. For product variety, we focused on a good or limited supply of different product types and strengths. Finally, provincial regulation was included so that we could see the relative importance of regulation in relation to other attributes and to explore what trade-offs customers may be willing to make. As an example of the variation between attribute levels, the attribute “proximity” consisted of four possible levels, in which the retailer was either within walking distance, within a 15-min drive, within a 30-min drive, or online with home delivery available.

**Table 1:** Attributes and levels for consumer experience

Attribute	Levels
**Prices**	Product discounts available Products offered at regular prices
**Product information available**	Only what is on the package Some additional information about the product Extensive information in each product such as terpene levels, grower, and supply chain information
**Customer service**	I can get all of my questions answered and can receive help selecting my products No one is available to answer questions of help select a product
**Proximity**	Within walking distance Store within a 15-min drive Store within a 30-min drive Online purchase with home delivery
**Product variety**	Limited product selection Wide product selection
**Store is provincially regulated**	Yes No Unknown

The DCE question was introduced by a preamble which described a scenario, to help give context to the consumer’s subsequent choice. The scenario description read as follows: “*You are going to make a cannabis purchase from a store either in person or online, which of the following locations would you choose? While some options may not seem possible, assume both options are available as presented.*”

In each choice task, consumers choose between two different combinations of attribute levels, which were described only by the arbitrary labels “Option A” or “Option B” [19]. A sample choice task can be found in the Supplemental Appendix. A fractional factorial design was applied. Six choice tasks were included in the analysis, allowing for a standard error below the 0.05 threshold. The survey included additional questions to gather sociodemographic data (e.g., age, province, sex, gender) as well as information about participants’ cannabis consumption and purchasing patterns.

### Analysis

Descriptive analysis was completed for sample characteristics. The DCE data were analyzed with a counts analysis, a multinomial logit (MNL) model, and a latent class model [20] using Sawtooth (Lighthouse Studio) software.

Average consumer preferences across the sample population were determined using the MNL model for the base analysis. Data were effects coded for each attribute except for cost, where continuous coding was used to allow for interpretable willingness to pay (WTP) values. Odds ratios were calculated using the least desirable level from each attribute as a reference point. WTP was calculated by estimating the marginal rate of substitution (MRS) by taking the ratio of two coefficients, with the linear cost estimate used for the comparison attribute.

Potential preference subgroups within the sample were identified using a latent class model. Differences in consumer characteristics across the groups were examined using segment membership probabilities estimated by Sawtooth. The significance of differences in participant characteristics between groups was tested using chi-squared tests.

### Ethical considerations

This study was carried out following the Tri-Council Policy Statement and was approved by the Memorial University Interdisciplinary Committee on Ethics in Human Research (File #20210143).

## Results

A total of 3261 individuals started the survey, of which 3181 consented, 1920 were eligible, and 1626 completed all questions. The most common reason for ineligibility was not having purchased cannabis in the past 12 months (*n* = 1240). Just over half (50.7%) of the sample identified as a man, and almost a third (31.5%) were between 30 and 39 years of age. There was a good representation of participants with respect to province of residence; however, the population was predominantly White (91.6%), and almost 90% had done some education beyond high school. With respect to frequency of cannabis use, 61.7% indicated they consumed cannabis at least once per week (Table 2).

**Table 2:** Sample characteristics

Characteristic	Number (%) N=1626
Sex	
Female	776 (47.8)
Male	833 (51.3)
Prefer not to say	14 (0.9)
Gender	
Woman	756 (46.5)
Man	825 (50.7)
Gender Diverse	17 (1.0)
Other	11 (0.6)
Prefer not to say	15 (0.9)
Age	
19-29	300 (18.5)
30-39	512 (31.5)
40-49	231 (14.2)
50-59	243 (14.9)
60 or above	340 (20.9)
Race	
Black	26 (1.6)
East/Southeast Asian	41 (2.5)
Latino	13 (0.8)
Middle	17 (1.0)
South Asian	32 (2.0)
White	1490 (91.6)
Other (please specify)	72 (4.4)
Province	
British Columbia	203 (12.5)
Alberta	207 (12.7)
Saskatchewan	155 (9.5)
Manitoba	142 (8.7)
Ontario	221 (13.6)
Quebec	206 (12.7)
New Brunswick	93 (5.7)
Nova Scotia	187 (11.5)
Prince Edward Island	28 (1.7)
Newfoundland and Labrador	173 (10.6)
Territories	11 (0.7)
Education	
Did not complete high school	23 (1.4)
High school diploma	141 (8.7)
Some post-secondary	237 (14.6)
College/trade/technical/ vocational training completed	533 (32.8)
Undergraduate degree	470 (28.9)
Graduate degree	222 (13.7)
Employment	
Full time student	123 (7.6)
Part time student	33 (2.0)
Unemployed, but seeking employment	71 (4.4)
Unemployed by choice	19 (1.2)
Unemployed due to disability	59 (3.6)
Employed part time	136 (8.4)
Employed full time	829 (51.0)
Self employed	144 (8.9)
Retired	287 (17.7)
Other (please specify:)	35 (2.2)
Income	
<$25,000	140 (8.6)
$25,000 to $49,999	314 (19.3)
$50,000 to $74,000	291 (17.9)
$75,000 to $99,999	264 (16.2)
$100,000 or more	486 (29.9)
Prefer not to say	130 (8.0)
Frequency of Cannabis purchase in last 12 months	
< 1 per month	824 (50.7)
1-2 times per month	514 (31.6)
3 or more times per month	288 (17.7)
Cannabis consumption frequency	
Less than once per month	354 (21.8)
At least once per month, less than once per week	259 (15.9)
At least once per week	322 (19.8)
Once per day	335 (20.6)
Multiple times per day	347 (21.3)
Prefer not to answer	9 (0.6)
Reason for cannabis use	
Medical (Self Prescribed)	222 (13.7)
Medical (Authorized)	106 (6.5)
Non-medical	629 (38.7)
Both medical and non-medical	650 (40.0)
Other	17 (1.0)
Initiation of Cannabis Use	
Since legalization	279 (17.2)
Used in the past then started again since legalization	603 (37.1)
Regular user prior to legalization	743 (45.1)
Cannabis Purchase Location	
Licensed in-person store	327 (84.9)
Licensed online store	174 (45.2)
Licensed Medical Dispensary	54 (14.0)
Unlicensed in-person store	66 (17.1)
Unlicensed online stores	113 (29.4)
Unlicensed connection on the community	106 (27.5)
Other	12 (3.1)

All attributes significantly influenced choice (*p* < 0.01) for the within-attribute chi-squared test. No attribute level dominated choices, with the level selection ranging from 38.8 to 61.0%. Significant between-attribute interactions were found between customer service and all other attributes examined.

The results of the MNL model showed that customer service carried the most weight in purchase decisions, followed by proximity and availability of sales and discounts. Product recommendations were the least relevant attribute (Table 3).

**Table 3:** Relative importance of retailer attributes on consumer decisions

	Label	Utility	Std error	Lower CI	Upper CI	OR
**Price**	Sales	0.26	0.01	0.234	0.285	1.68
	No sales	− 0.26	0.01	− 0.285	− 0.234	Ref
**Product Information**	Package	− 0.14	0.02	− 0.175	− 0.096	Ref
	Some extra	− 0.08	0.02	− 0.117	− 0.039	1.06
	Extensive	0.21	0.02	0.174	0.254	1.42
**Customer service**	Good	0.34	0.01	0.316	0.368	1.98
	Poor	− 0.34	0.01	− 0.368	− 0.316	Ref
**Proximity**	Walk	0.29	0.03	0.237	0.337	1.22
	15 min	0.11	0.03	0.058	0.156	1.02
	30 min	− 0.48	0.03	− 0.532	− 0.430	0.57
	Online	0.09	0.02	0.038	0.135	Ref
**Product variety**	Limited	− 0.18	0.01	− 0.206	− 0.155	Ref
	Wide	0.18	0.01	0.155	0.206	1.44
**Store is provincially regulated**	Yes	0.28	0.02	0.236	0.315	1.59
	No	− 0.19	0.02	− 0.224	− 0.147	Ref
	Unknown	− 0.09	0.02	− 0.128	− 0.052	1.10

*Model* Multinomial logit model, *CI *Confidence interval, *OR *Odds ratio

A five-group latent class model demonstrated the best fit (Table 4). Group 1 prioritized customer service (24.9% of sample), group 2 prioritized proximity with a preference towards brick-and-mortar stores (14.6%), group 3 prioritized detailed product information (21.5%), group 4 prioritized provincial regulation (14.9%), and group 5 prioritized product sales and discounts (24.1%). Interestingly, only one group placed a high value on the store being provincially regulated, while three groups were willing to make a trade-off with regulation to access better customer service or closer proximity. Only group 5 indicated that they preferred non-regulated stores over regulated ones (Table 5).

**Table 4:** Latent class model fit statistics

Groups	CAIC	BIC
2	11,622.03	11,601.03
3	11,536.95	11,504.95
4	11,511.20	11,468.20
5	11,503.51	11,449.51

*CAIC *Consistent Akaike information criterion, *BIC *Bayesian information criterion

**Table 5:** Relative importance of retailer attributes on consumer decisions by latent class

	Segment size	Group 1–24.9%		Group 2–14.6%		Group 3–21.5%		Group 4–14.9%		Group 5–24.1%	
		**Effect estimate**	**OR**	**Effect estimate**	**OR**	**Effect estimate**	**OR**	**Effect estimate**	**OR**	**Effect estimate**	**OR**
**Prices**	Sales	0.18	1.44	0.51	2.78	0.20	1.50	0.24	1.63	0.58	3.17
	No sales	− 0.18	Ref	− 0.51	Ref	− 0.20	Ref	− 0.24	Ref	− 0.58	Ref
**Product information available**	Package	− 0.58	Ref	− 0.29	Ref	− 0.31	Ref	− 0.20	Ref	0.06	Ref
	Some extra	− 0.22	1.43	− 0.09	1.22	− 0.09	1.26	− 0.18	1.02	− 0.02	0.93
	Extensive	0.79	3.92	0.37	1.93	0.40	2.04	0.38	1.79	− 0.04	0.91
**Customer service**	Good	1.23	11.67	0.30	1.82	0.43	2.37	0.46	2.49	0.02	1.04
	Poor	− 1.23	Ref	− 0.30	Ref	− 0.43	Ref	− 0.46	Ref	− 0.02	Ref
**Proximity**	Walk	0.09	1.23	1.65	27.31	1.07	0.85	− 0.10	0.83	− 0.21	0.56
	15 min	0.04	1.16	0.78	11.50	− 0.05	0.28	0.29	1.22	− 0.01	0.68
	30 min	− 0.01	1.11	− 0.77	2.45	− 2.26	0.03	− 0.28	0.70	− 0.16	0.59
	Online	− 0.12	Ref	− 1.66	Ref	1.23	Ref	0.09	Ref	0.38	Ref
**Product variety**	Limited	− 0.33	Ref	− 0.24	Ref	− 0.32	Ref	− 0.07	Ref	− 0.25	Ref
	Wide	0.33	1.92	0.24	1.61	0.32	1.91	0.07	1.14	0.25	1.63
**Store is provincially regulated**	Yes	0.35	1.77	0.33	1.89	0.11	1.23	2.33	49.67	− 0.16	0.78
	No	− 0.22	Ref	− 0.31	Ref	− 0.10	Ref	− 1.58	Ref	0.08	Ref
	Unknown	− 0.13	1.10	− 0.02	1.34	− 0.01	1.09	− 0.75	2.28	0.08	1.00

*OR *Odds ratio

Both groups 1 and 2 preferred to shop from brick-and-mortar stores; however, group 2 placed a much higher value on proximity. Three groups (groups 3, 4, and 5), representing 60.5% of the sample, preferred online stores. Retailer proximity was more important than most other attributes for 60% of our sample (groups 2, 3, and 5). These preferences contrasted with the actual licensed purchase behavior described in the survey, where 85% reported making some purchases through licensed in-person stores and 45% through licensed online stores. The sample’s preferences for online versus brick-and-mortar stores were more in line with unlicensed purchase behavior, as 17% and 29% of the sample reported shopping from unlicensed in-person and online stores, respectively.

**Fig. 1:**
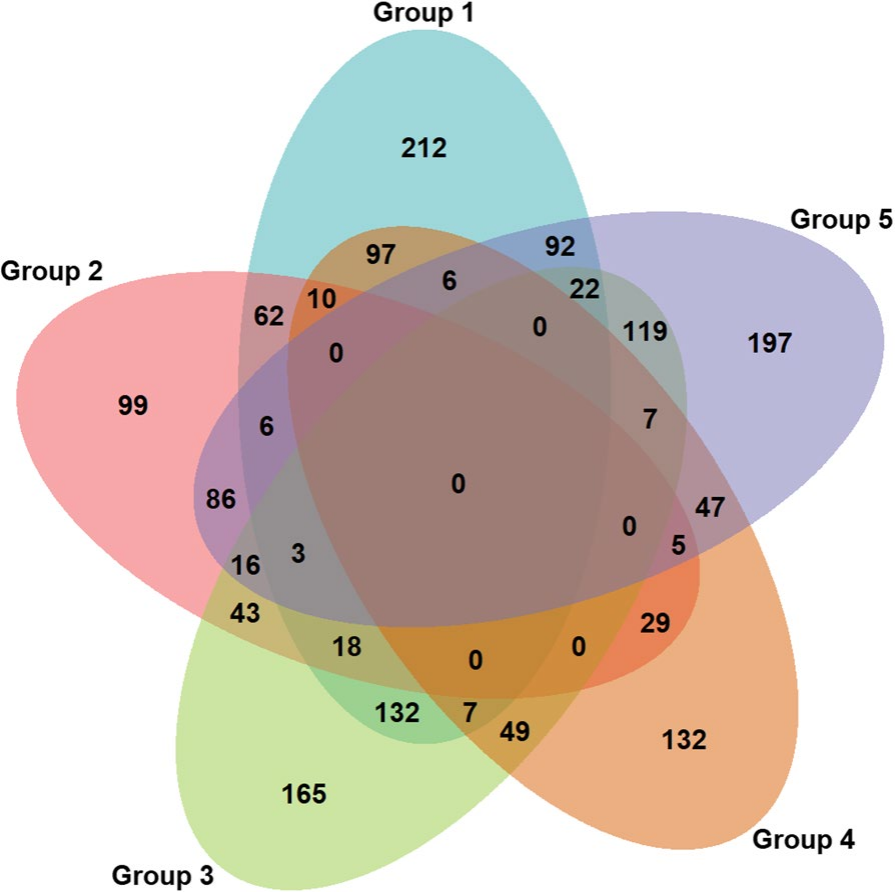
Venn diagram of group membership for latent class model. This Venn diagram depicts the number of individuals that fall within each latent class represented by different colored ovals. Overlapping segments of the ovals depict respondents who exhibit characteristics of one or more of the different latent groups

The Venn diagram (20% inclusion) shows that while there were individuals who had preference tendencies that were represented in two unique groups from the latent model, very few had tendencies that were in line with three or more groups (Fig. 1).

The distribution of group membership by age, sex, and cannabis use history (including purpose, frequency, amount, and if they started consuming prior to legalization) each significantly impacted group membership (Table 6). On inspection of group membership plots (Supplementary Appendix), groups 2 and 5 (the groups with a higher preference for sales) were more likely to be male, and to purchase and consume cannabis more frequently and purchase larger amounts. Those in groups 1 and 4 (who placed a higher preference for regulated status and availability of information) were more likely to have started consuming cannabis after legalization.

**Table 6:** Latent class significance of group membership by participant characteristic

Factor	Chi-squared	*p*-value
Age	28.91	0.025
Sex	22.69	0.004
Province	60.32	0.109
Income	23.27	0.276
Cannabis use in the past 12 months	29.43	0.000
Frequency of cannabis use	61.31	0.000
Amount of cannabis use	35.08	0.000
Purpose of cannabis use	45.67	0.000
Use of cannabis pre-legalization	77.74	0.000

## Discussion

This study demonstrated that customer service ranked as the most important attribute for the sample as a whole; however, there was considerable heterogeneity in terms of consumer preferences for cannabis retailer characteristics, as shown by the latent class model. Several preference patterns exist, and data from our study can help to better understand sub-groups of the cannabis consumer population to inform policies that impact how cannabis is sold in Canada.

While customer service was the most important attribute for the entire sample, this result was driven by two sub-groups (groups 1 and 4) that placed a very high priority on this characteristic. Group membership analysis showed that individuals in these groups were more likely to be those who purchased and consumed less frequently and those who started consuming after legalization. Interestingly, members of these sub-groups also placed particular importance on store regulation and the availability of extensive product information. The importance of customer service and product knowledge was supported by our previous work that consumers looked to cannabis store staff for information on the potential effects of a particular product, such as whether it would be relaxing, energizing, or could reduce pain, as well product lineage [9]. Other literature has shown that customers are comparing the customer service they get from regulated stores from what they are used to in the legacy market [21].

Consumer preference for customer service presents a challenge for licensed cannabis vendors, as retail regulations limit the extent to which cannabis store staff can share knowledge with consumers. The Cannabis Act [1] states that a person authorized to sell cannabis can only promote a product at the point of sale in terms of its price or availability, prohibiting any testimonials or personal endorsements of products. This means that the customer service offered by staff in regulated stores, if adhering to policy, is extremely limited. In some provinces, access to customer service and product information varies by store type. In Newfoundland and Labrador, the private cannabis retail model includes four tiers of cannabis stores [22]. Tiers 1 and 2 include standalone stores that prevent the entrance of minors and can only share information as outlined in the Cannabis Act [1]. Tier 3 and 4 stores allow the sale of other products, and minors are permitted on the premises. Staff in these stores can share government approved information through weblink and pamphlets, but it cannot be verbally shared between retailer and customer. It has also been noted that staff within publicly run stores are not necessarily hired based on their cannabis knowledge or ability to support customers in product selection [9]. These factors may deter consumers who place a high value on customer service from shopping for cannabis in regulated stores, particularly if they have access to knowledgeable sources elsewhere.

Product variety was moderately important for four out of five sub-groups in the current study, as indicated by consumers’ willingness to make trade-offs with other attributes in favor of a store with more selection. One attribute that was frequently traded off in favor of greater product variety was store regulation. This finding is in line with data from the 2021 Canadian Cannabis Survey [2] which found that of respondents that had used cannabis in the last 12 months, 55% reported having intended to purchase a product from a regulated source only to find that it was unavailable. This is less of an issue for dried flower cannabis or cannabis oils, but with the strict limitation of 10 mg of THC per package of cannabis edibles [1], many customers were not able to get their desired products from licensed stores. Additionally, some provinces have banned sales of all cannabis vapes due to the emergence of EVALI [23], making them only accessible to individuals in these provinces who have medical authorization. Limited variety and restrictions on product type and potency at regulated retailers may cause consumers to continue purchasing cannabis from unregulated sources.

Despite the perseverance of the unregulated cannabis market, purchasing a regulated product was preferred by most consumers. However, only 15% of the consumer sample placed Health Canada regulation status as the most important purchasing factor, as other groups were willing to trade off regulation in favor of proximity, customer service and sales. One issue with regulated cannabis stores is limited access, both in terms of proximity and hours of operation. With this in mind, privately run and hybrid retail models tend to have more cannabis stores per capita, with longer hours of operation than government-run retail models [24]. The increased accessibility to regulated cannabis products within provinces with private and hybrid retail models may be better positioned to strengthen legal sales.

There are mixed opinions on the value of increased public access to retail cannabis stores. On one hand, reducing access to cannabis retail stores may be an effective way to reduce consumption and related harms [24]. Meanwhile, increased access to regulated cannabis retail shops is an essential step towards decreasing unregulated sales. This perspective was supported by the literature, as Wadsworth et al. [25] found a positive relationship between the proximity of a regulated cannabis store and the likelihood of a respondent’s most recent cannabis purchase having been legal. For consumers who place a high value on proximity and convenience, a lack of legal storefronts may encourage them to obtain cannabis illegally. Provinces took a variety of approaches to spread out access to retail cannabis stores. For example, in Newfoundland and Labrador, retailer cannabis licenses are distributed based on postal code, to ensure widespread access while also not over saturating in some areas [26]. Other provinces, such as Manitoba and Ontario, have municipalities determine if a retail cannabis store will be allowed in their jurisdictions; however, limitations on store density do not seem to exist [27, 28]. In provinces that operate under a solely public retail model, the retail locations are determined by the province. There is an opportunity to do further research on these differences in store density and the impact it has on consumer decisions and behaviors.

Availability of sales and discounts was ranked as more important than most other attributes for about 39% of our sample (groups 2 and 5) and as the most important for 24%. Analysis of group membership data revealed that these groups also were more likely to be frequent purchasers and consumers, making up a large proportion of the cannabis market. Legal cannabis prices remained higher than those of the unlicensed market due to the expenses associated with extensive product testing and approval [9]. In a 2020 study, Mahamad et al. reported that legal dried flower cannabis was on average 19% more expensive than unregulated dried flower cannabis, noting that the price disparity increased with larger quantity purchased [29]. The Cannabis Act outlines maximum quantities that a person can purchase or possess at any time as well as strict regulations preventing any marketing or promotion activities such as discounts or bulk purchase offers [1]. These regulations prevent licensed stores from selling cannabis in quantities or at the price points that can be accessed through unlicensed sources. While this price disparity may deter some consumers, others may be willing to pay more for legal cannabis. Amlung and MacKillop found that consumers perceived legal cannabis as superior and preferred it when priced the same as or even slightly higher than unregulated cannabis [30]. However, once the price of legal cannabis passed a certain threshold, the unregulated product was preferred. Unless regulated cannabis stores can find a way to compete with unregulated prices, consumers primarily concerned with product cost may continue to buy from unregulated sources [29, 30].

There are several limitations to this study. Those limitations that are inherent to discrete choice studies, including ordering effect, hypothetical bias, and framing effect [14], are discussed along with the methods used to mitigate against them in the methods supplement of a previously published paper [17]. One attribute that was important when considering retailer preferences was store ambiance, as was noted in our preliminary qualitative study [9] and other retail preference studies [10]. Comments we have heard related to public stores suggested that these venues lack character. Meanwhile, private stores provide a variety of different atmospheres, with some catering to “stoner” culture and others displaying very sleek and minimalistic decor. It was not possible to include elements of store ambiance in this DCE as words could not adequately describe this attribute, and images would reveal other store characteristics that could influence choices. In future research, it would be interesting to look at the impact of ambiance on consumer behavior and see if consumers with certain characteristics were drawn to different store types. Another limitation of this research involved the demographic makeup of the participants. In the current study, 29.9% of participants reported an annual income of $100,000 or more. In comparison, the Canadian Census [31] reported that only 10.8% of 2021 respondents had an annual income of $100,000 or more. With this considered, the participant group in the present study was substantially more affluent than the general population of Canada, which may interfere with the ability to generalize findings to all Canadian consumers. Future research should explore whether less financially stable consumers would be willing to make the same price trade-offs in favor of other purchasing factors. Finally, consumer decisions on where to make purchases are also influenced by the characteristics of the products within the store. Product attributes (e.g., potency, packaging) could not be brought into this DCE; however, they were explored within the survey [16, 17]. To truly understand consumer choices, you need to consider both the characteristics of the products and retailer.

## Conclusion

This study found that there is considerable heterogeneity in preference patterns for cannabis retailers among consumers. Overall, customer service was the most important factor in retailer selection and product information. However, consumers who purchase and consume more frequently focus primarily on the availability of sales and easy accessibility and less on the regulated status of the store. These findings can inform efforts to attract more buyers to the legal market. Given the preferences expressed by participants, such efforts should involve greater access to customer service, detailed product information, and competitive pricing including sales and discounts.

### Supplementary Information

**Additional file 1.** 

## Data Availability

Data can be made available upon request and with the approval of the Interdisciplinary Committee on Ethics in Human Research. **Code availability** Not applicable.

## Abbreviations

CBD: Cannabidiol
DCE: Discrete choice experiment
EVALI: E-cigarette- or vaping-use-associated lung injury
MNL: Multinomial logit
MRS: Marginal rate of substitution
THC: Tetrahydrocannabinol
WTP: Willingness to pay
