# Supplementary material for: Examining attributes of retailers that influence where cannabis is purchased: a discrete choice experiment
Source: J Cannabis Res. 2024 Feb 8;6:4. doi: 10.1186/s42238-023-00204-w (PMC10851494; doi:10.1186/s42238-023-00204-w)

**Examining attributes of retailers that influence where cannabis is purchased: A discrete choice experiment**

Supplementary Appendix


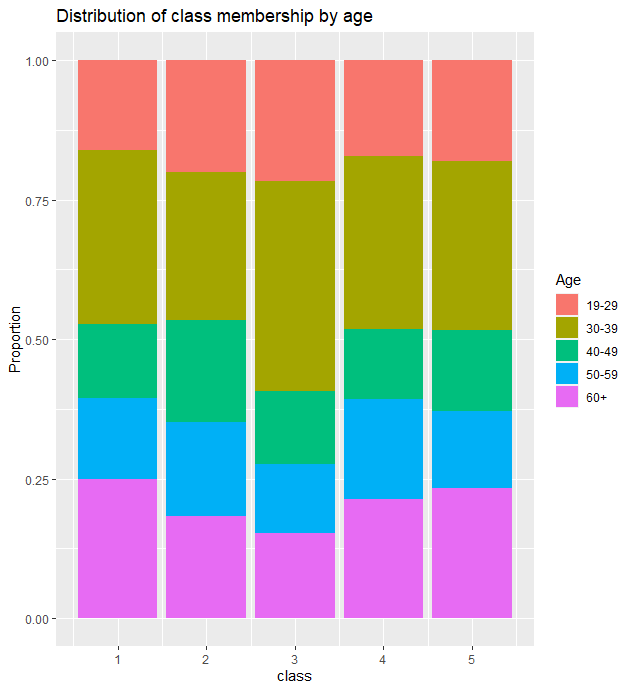


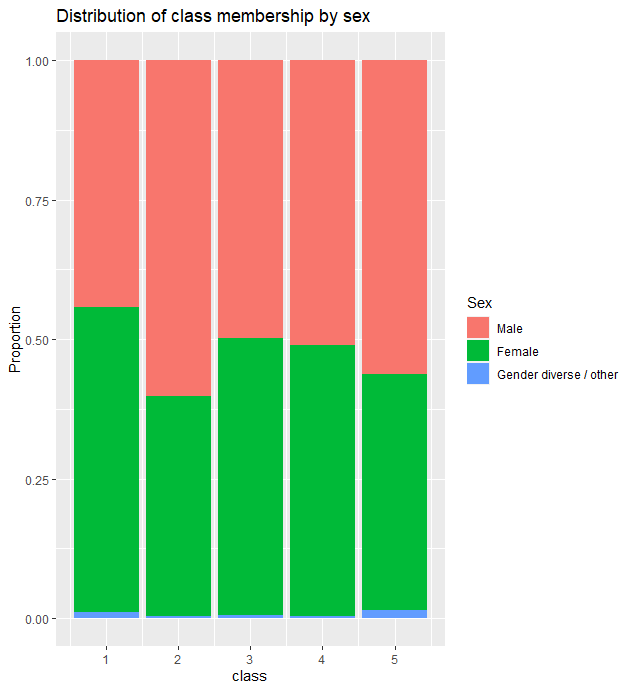


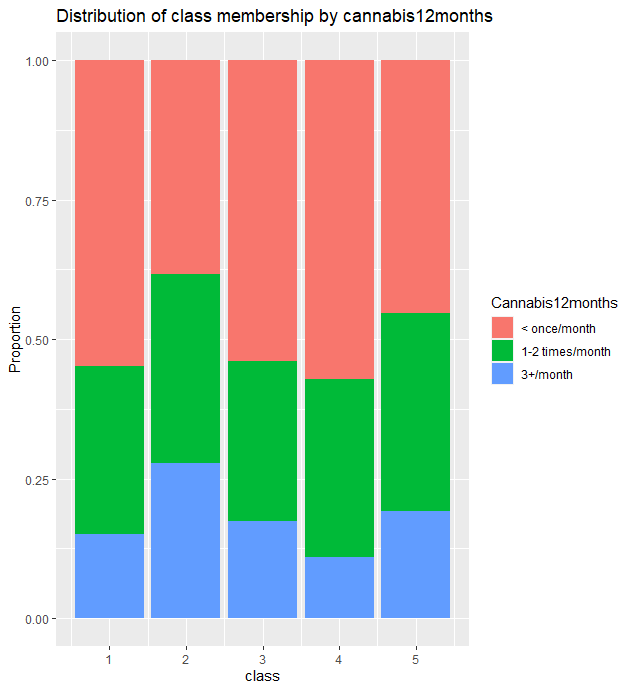


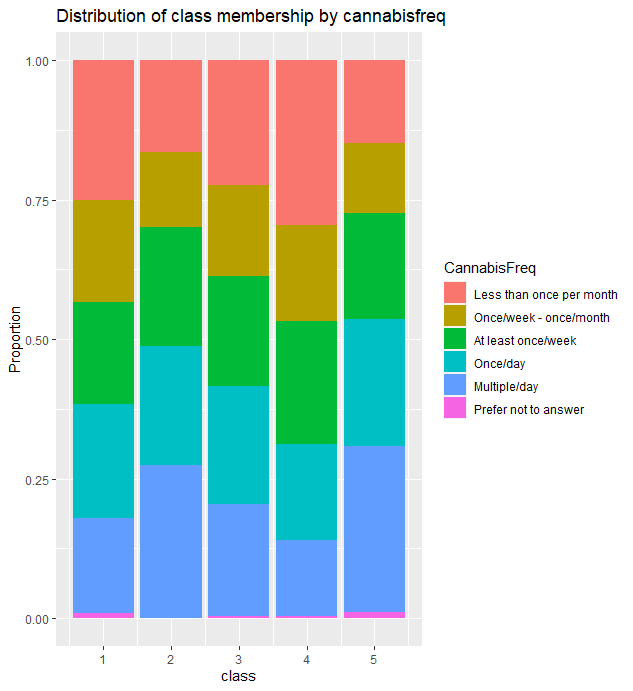


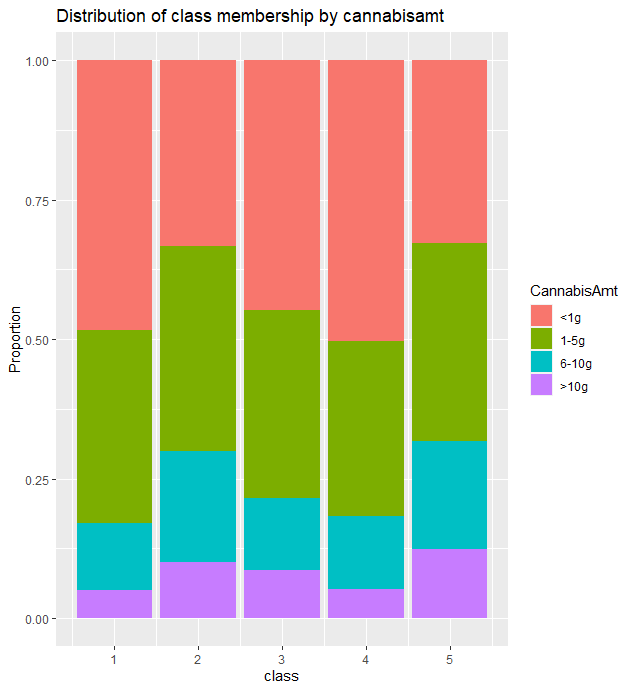


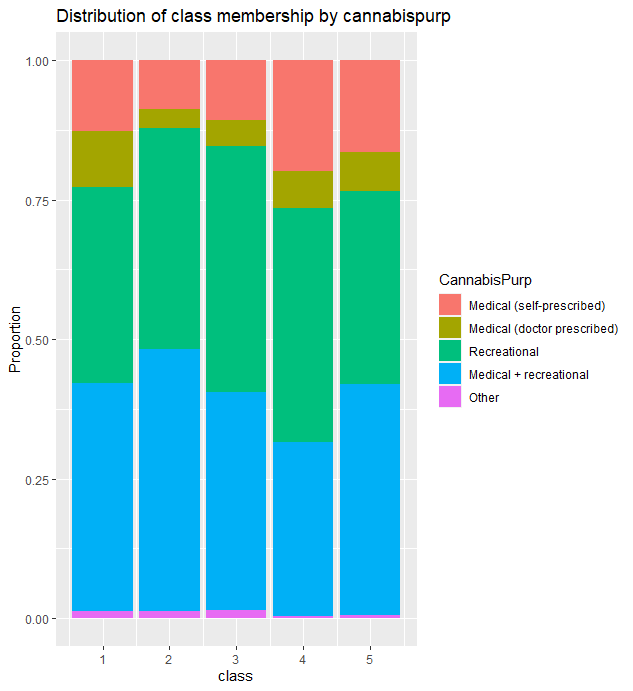


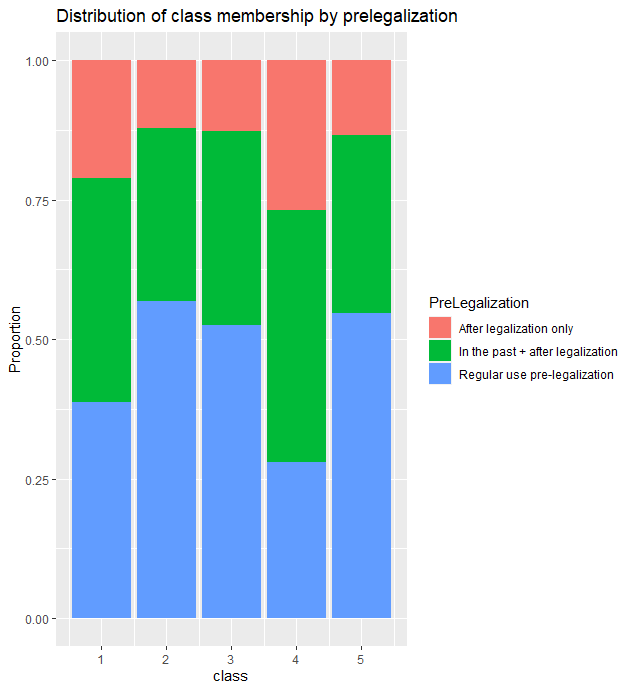


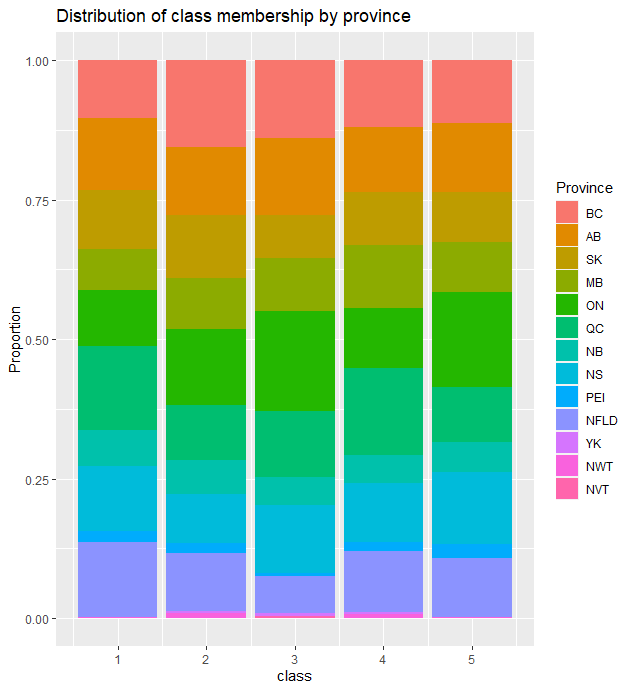


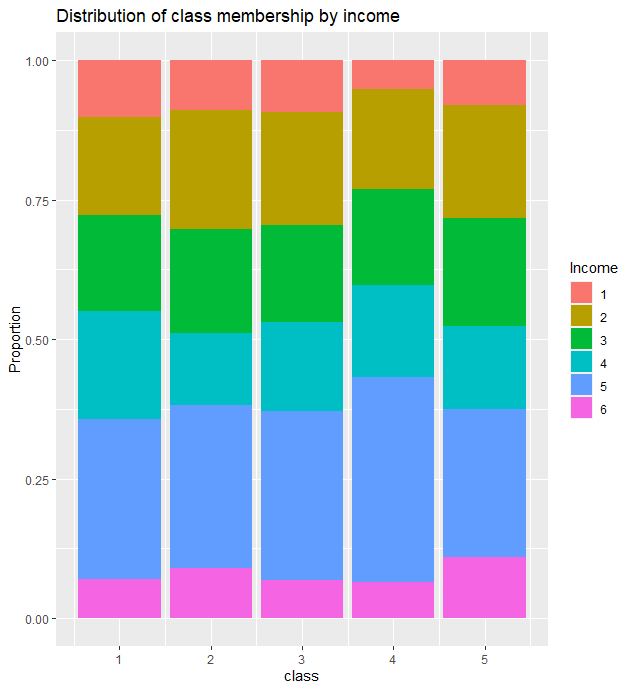

Supplement: Supplementary file 1 — Additional file 1. [file 42238_2023_204_MOESM1_ESM.docx]
